# Investigation of the effect of structure modification of furamidine on the DNA minor groove binding and antiprotozoal activity

**DOI:** 10.1016/j.ejmech.2023.115287

**Published:** 2023-04-05

**Authors:** Abdelbasset A. Farahat, Arvind Kumar, Tanja Wenzler, Reto Brun, Ananya Paul, Pu Guo, W. David Wilson, David W. Boykin

**Affiliations:** a Masters of Pharmaceutical Sciences Program, California Northstate University, Elk Grove, CA, 95757, USA; b Department of Pharmaceutical Organic Chemistry, Faculty of Pharmacy, Mansoura University, Mansoura, 35516, Egypt; c Department of Chemistry, Georgia State University, Atlanta, GA, 30303, USA; d Swiss Tropical and Public Health Institute, Basel, 4002, Switzerland; e University of Basel, Basel, 4003, Switzerland

**Keywords:** Amidines, Stille coupling, Lithium bis(trimethylsilyl)amide, DNA minor Groove binders, Antimalarial, Antitrypanosomal

## Abstract

New analogs of the antiprotozoal agent Furamidine were prepared utilizing Stille coupling reactions and amidation of the bisnitrile intermediate using lithium bis-trimethylsilylamide. Both the phenyl groups and the furan moiety of furamidine were replaced by heterocycles including thiophene, selenophene, indole or benzimidazole. Based upon the Δ*Tm* and the CD results, the new compounds showed strong binding to the DNA minor groove. The new analogues are also more active both *in vitro* and *in vivo* than furamidine. Compounds 7a, 7b, and 7f showed the highest activity *in vivo* by curing 75% of animals, and this merits further evaluation.

## Highlights

• Twelve new diamidine compounds were made.
• Δ*Tm* and the CD data were acquired. The new compounds showed strong binding to the DNA minor groove.
• The new compounds were tested *in vitro* against both STIB900 of *T. b. r.* and *P. f.* (*Plasmodium falciparum*) strain*.* K1.
• *In vivo* efficacy was determined in *T. b. rhodesiense* (STIB900) infected mice at 4 × 5 mg/kg.
• Compounds 7a, 7b, and 7f showed the highest activity *in vivo* by curing 75% of animals.

## Graphical abstract

**Figure:**
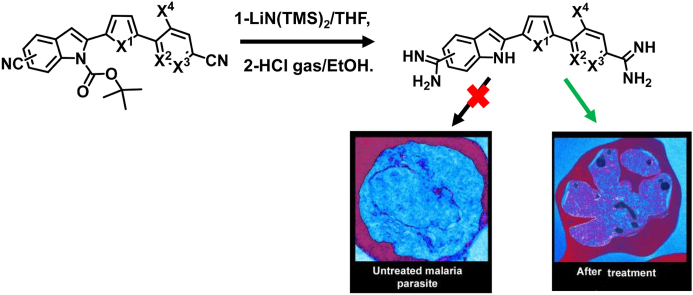

## 1 Introduction

**Fig. 1:**
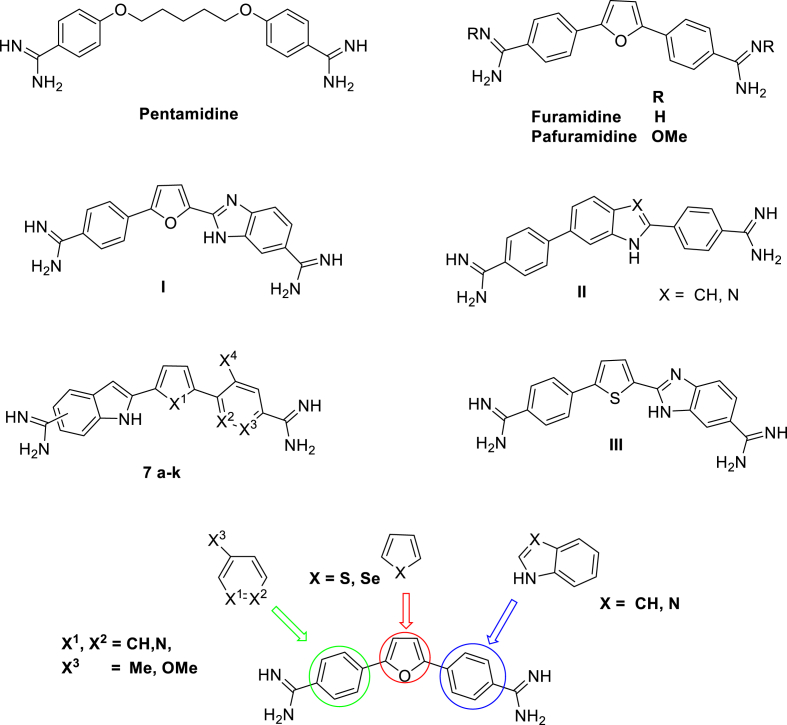
Structure of active antiparasitic diamidines and their modification.

Malaria is a lethal infectious disease that transmitted by the bite of an infected female *Anopheles* mosquito. After infection the patient starts suffering from fever, headache, tiredness and vomiting and if not treated it leads to death. According to the WHO, Globally in 2021, there were an estimated 247 million malaria cases in 84 malaria endemic countries [1]. Drugs are available to treat malaria but drug resistance is a big issue even to the highly active artemesinin based combination therapy (ACT) [2]. Without an effective drug, Malaria is lethal whereas resistance develops against the current drugs. Trypanosomiasis (sleeping sickness) is another neglected infectious disease that transmitted via the blood feeding tsetse flies. More than 65 millions around the world are subject to infection within 36 countries [3]. The parasite first invades the blood and lymph system then attacks the central nervous system [4] and if not treated it leads to death. The available drugs that treat Trypanosomiasis still unsatisfactory due to difficult administration, severe side effects and emergence of resistance [5]. Now, it is crucial to develop new potent and more tolerated drugs to treat these infectious diseases. Diamidines containing hetyerocycles that bind in the DNA minor groove, showed activity against both Malaria and Trypanosoma. Pentamidine (Fig. 1) is the drug of choice for treating *T. b. gambiense* since 1930 [6]. Diminazene, another diamidine is being used to treat animal trypanosomiasis since 1950 [7]. Furamidine (Fig. 1) made by our group showed more potency and less toxicity than pentamidine against trypanosomiasis [8]. Pafuramidine (Fig. 1), is the orally active form of furamidine showed excellent activity against both sleeping sickness and malaria in phase II clinical trials [9], but due to the discovery of liver and kidney toxicity in few volunteers, the pafuramidine development was ended [8]. In a previous investigation we have replaced one of the phenyl ring of furamidine with benzimidazole (compound I, Fig. 1), this approach resulted in more active compound *in vivo* than furamidine with comparable DNA binding affinity of furamidine [10]. In another investigation we explored the replacement of the linker furan ring between the diphenyl rings of furamidine with both benzimidazole and indole rings (comp II, Fig. 1) [11]. In general, this linker change enhanced both the DNA binding and the antiparasitic activity of the new diamidines in comparison with furamidine.

Aiming for making more potent and less toxic antiprotozoal agents, we designed and synthetized new analogues of both furamidine and compound I. In the current investigation, we modified the structure of furamidine by replacing one of the phenyl rings with indole while keeping the other phenyl as it is or replacing it also with aryl or heteroaryl ring (comps 7a-k, Fig. 1). This approach is considered also as a direct modification of compound I via replacing the benzimidazole ring with an indole ring. Moreover, we modified the structure of compound I by replacing the central furan linker with thiophene (comp III, Fig. 1).

## 2 Results and discussion

### 2.1 Chemistry

**Scheme 1:**
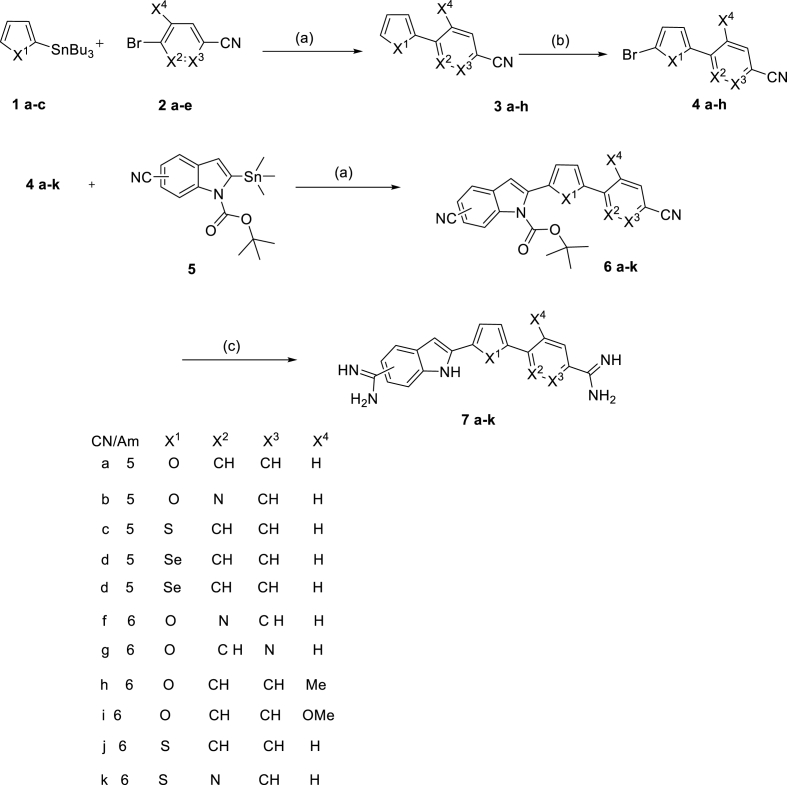
Reagents and conditions; (a) pd(pph_3_)_4_, Dioxane; (b) NBS/DMF; (c) i-LiN(TMS)_2_/THF, ii-HCI gas/EtOH.

Synthesis of the indole diamidines 7a-k is mentioned in Scheme 1. The bromoaryl or heteroarylnitriles 2a-d were allowed to react with the tin compounds 1a-c by applying Stille coupling conditions [12] using Pd (0) as catalyst in dioxane to afford the intermediates 3a-h. Bromination of 3a-h using NBS in DMF [13] afforded the bromo analogues 4a-h. The indole stannane 5 was allowed to couple with the bromo intermediates 4a-h utilizing Stille coupling reaction to produce the bisnitriles 6a-k in good yield (61–77%). The final diamidines 7a-k were produced by reaction of the bisnitriles with lithium bis(trimethylsilyl)amide in THF [14], and then treating the silylated intermediate with ethanolic HCl.

Compound III was prepared according to the published procedures [15].

### 2.2 Biology

Table 1 contains the screening data for the new diamidines and the data for both furamidine and compound I are included for comparison aims. It was reported by us that the difference in increase of the melting temperature (Δ*Tm)* between the poly (dA-dT) DNA/compound complex and the free DNA gives a useful method for determining the binding affinities for the diamidines [16,17]. The mechanism of antiparasitic action of the diamidines is attributed to inhibition of the DNA transcription or interaction with DNA dependant enzymes [18]. We observed that the new diamidines bind strongly to the DNA as indicated from the high Δ*Tm* of the poly (dA-dT)/diamidine complex that ranges from 27 °C to more than 30 °C.

**Table 1:** DNA affinities and antiparasitic activity for the novel diamidines.

	ΔTm (^o^C)^a^	T. b. r. (nM)^b^	P.f. (nM)^c^, ^d^	Cytotox. (μM)^d^	SI/T. b. r.^e^	SI/P.f^f^	In vivo (cures)^g^
	25	4.5	15.5	6.7	1488	432	1/4
	25	122	95.8	10.1	82	105	1/4
	>30	27	11.8	22.4	829	1898	3/4
	29	2	2.8	14.2	7100	5071	3/4
	27	6	5.8	50.4	8400	8689	3/4
	29	6	2.8	17.0	2833	6071	2/4
	>30	3	3.9	21.5	7166	5512	2/4
	>30	12	4.6	12.9	1075	2804	0/4
	28	17	6.4	34.8	2047	5437	3/4
	28	7	3.6	14.2	2028	3944	0/4
	>30	6	ND	5.1	850	ND	0/4
	27	39	ND	11	282	ND	ND
	>30	12	7.2	12.4	1033	1722	0/4
	29	15	ND	14.5	966	ND	0/4

a Thermal melting increase of poly(dA-dT)_n_.

b STIB900 was the strain of *T. b. r*. (*Trypanosoma brucei rhodesiense*) used.Values are the average of duplicate determinations.

c IC_50_ values obtained against the chloroquine resistant *P. f*. (*Plasmodium falciparum*) strain. K1. Values are the average of duplicate determinations.

d Cultured L6 rat myoblast cells was used for cytotoxicity assessment.

e Selectivity Index for *T. b. r*. is the ratio: IC_50_ (L6)/IC_50_ (*T b.r*.).

f Selectivity Index for *P. f*. is the ratio: IC_50_ (L6)/IC_50_ (*P.f*.).

g In vivo efficacy determined in *T. b. rhodesiense* (STIB900) infected mice at 4 × 5 mg/kg i.p. dose except 293 at 4 × 20 mg/kg and 818 at 4 × 10 mg/kg.

**Fig. 2:**
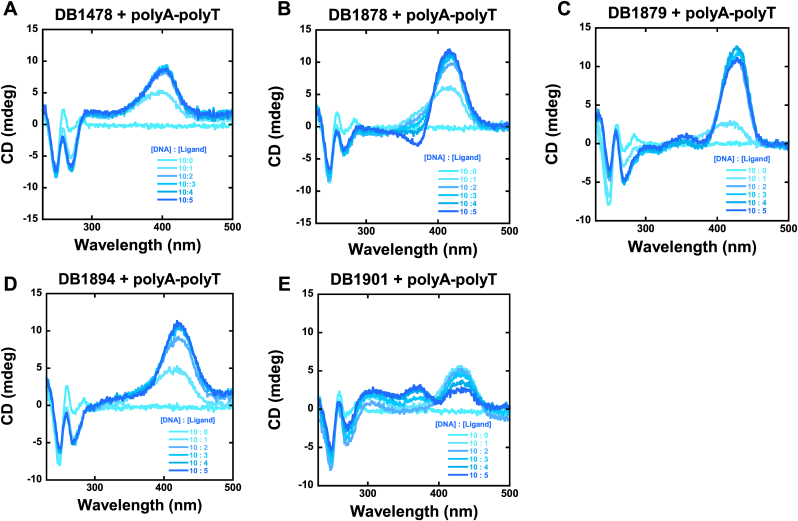
Circular dichroism spectra for the titration of representative compounds, DB1478 (7a) (A), DB1878 (7e) (B), DB1879 (7j)(C), DB1894 (7f) (D), and DB1901 (7d) (E) with a 20 μM polyA-polyT sequence in Tris-HCl buffer (50 mM Tris-HCl, 100 mM NaCl, 1 mM EDTA, pH 7.4) at 25 °C.

CD titration experiments are a convenient and effective tool of evaluating the binding mode and the saturation limit for compounds binding with DNA sequences. CD spectra monitor the asymmetric environment of the compounds binding to DNA and therefore can be used to obtain information on the binding mode [19,20]. The CD spectra for the interaction between poly (dA-dT) and five of the diamidines (7a, 7d, 7e, 7f, 7j) was observed. There are no CD signals for the free compounds but on the addition of the compounds into DNA, substantial positive induced CD signals (ICD) rose in the absorption range between 360 and 470 nm. These positive ICD signals indicate that the selected ligands are a minor groove binders, as expected from their chemical structures. As can be seen from Fig. 2, all tested compounds form complexes that are minor groove binders of the polyA-polyT sequences. In summary, the CD titration results confirm a minor groove binding mode for the tested compounds.

The new benzimidazole and indole diamidines III, and 7 a-k showed excellent *in vitro* activity against the long slender blood stream forms of *T. b.r.*. (IC_50_s ranges from 2 to 39 nM) and are also very potent against *Plasmodium falciparum* (IC_50_s range from 2.8 to 13.2 nM). From this excellent *in vitro* data, we conclude that replacing one of the furamidine phenyls with indole (7a-k) gave more potent compounds against both parasites, also replacing the furan ring of compound I with thiophene (III) gave highly active compounds. Except for 7g activity against *T. b. r.*, all compounds that have pyridine substituents are less active than their phenyl counterparts against both parasites.

All the newly synthetized compounds exhibited excellent safety by having high selectivity against both parasites, the selectivity indices range from 829 to 8400 against *T. b. r.* and from 1722 to 8689 against *P. f.* and these selectivity indices are higher than that of both furamidine and compound II.

After monitoring the potent *in vitro* activity and selectivity of the new indole and benzimidazole diamidines, we decided to test them *in vivo* in the rigorous *T. b. r*. STIB900 mouse model (Table 1) against the acute hemolymphatic stage of the T. b. r. The animals were injected with a daily intraperitoneal (i.p.) dose of 5 mg/kg compound for four consecutive days (except compound III the dose was 10 mg/kg). At this dose both furamidine and compound I cures only 25% of the infected mice. Compounds III, 7a, 7b, and 7f showed potent activity by curing 75% of animals while compounds 7c and 7d showed moderate activity by curing 50% of the animals and the rest of compound were inactive *in vivo*. This potent activity of III, 7a, 7b, and 7f suggests further evaluation.

## 3 Conclusion

In this project we modified furamidine structure with three different approaches, either by replacing one phenyl ring with indole or benzimidazole, or replacing the furan spacer with thiophene or selenophene or replacing the other phenyl ring with aryl or heteroaryl ring. All these modifications resulted in stronger DNA minor groove binders as seen from the high Δ*Tm* and changes in the CD). The new analogues are also more active both *in vitro* and *in vivo* than both furamidine and pentamidine. Compounds III, 7a, 7b, and 7f showed the highest activity *in vivo* by curing 75% of animals, and this merits further evaluation.

## 4 Experimental

### 4.1 Biology

#### 4.1.1 *In vitro*, *In vivo* efficacy and cytotoxicity studies

The *in vitro* screening was done against the *T. b. r*. strain STIB 900 and the chloroquine resistant *P. f*. strain K1. The *in vivo* studies were done with the STIB900 acute mouse model for *T. b. r.* infections [21]. Dosage was 4 × 5 mg/kg i.p. (except for III it was 4 × 10 mg/kg i.p) performed as described before in detail [22]. Cytotoxicity was determined in L6 rat myoblast cells as previously reported in detail [23].

#### 4.1.2 Thermal melting (TM) measurements

Thermal melting experiments were performed using a Cary 300 spectrophotometer. Cuvettes were mounted in a thermal block while the solution temperatures were mesaured with a thermistor in the reference cuvette. Temperatures were maintained under computer control and increased at 0.5 ^°^C/min. The experiments were performed in 1 cm path length quartz cuvettes in CAC 10 buffer (cacodylic acid 10 mM, EDTA 1 mM, NaCl 100 mM with NaOH added to give pH = 7.0). Poly (dA-dT) DNA concentrations were recorded by measuring its absorbance at 260 nm. A ratio of 0.3 mol compound per mole of DNA was used for the complex and DNA alone was used as control [18]. ΔTm values were determined from the peak of first derivative curves (dA/dT).

#### 4.1.3 Circular dichroism (CD)

Circular dichroism experiments were performed on a Jasco J-1500 CD spectrometer in 1 cm quartz cuvette at 25 °C. A buffer scan as a baseline was collected first in the same cuvette and subtracted from the scan of the following samples. The hairpin DNA sequence polyA-polyT (20 μM), in TNE 100 was added to the cuvette prior to the titration experiments, and then the compound was added to the DNA solution and incubated for 10 min to achieve equilibrium binding for the DNA-ligand complex formation. For each titration point, four spectra were averaged from 500 to 230 nm wavelength with a scan speed of 50 nm/min, with a response time of 1 s. Baseline-subtracted graphs were created using the KaleidaGraph 4.0 software [24].

### 4.2 Chemistry

All commercial reagents were used without further purification. All melting points were determined on an original Mel-Temp 3.0 melting point instrument, and are uncorrected. TLC was carried out using silica gel 60 F254 precoated aluminum sheets using UV light for detection. ^1^H and ^13^C NMR spectra were recorded on a Bruker 400 MHz spectrometer using the indicated solvents. Mass spectra were recorded at the Georgia State University Mass Spectrometry Laboratory, Atlanta, GA. If the compounds are reported as salts contain water and/or solvents, these components were detected in HNMR spectra. Elemental analysis were determined by Atlantic Microlab Inc., Norcross, GA. Compounds 6a,7a [25] and 6e,7e [26] were reported.

#### 4.2.1 Synthesis of 2-(4-Cyanoaryl)furan, thiophene or selenophene (3 a-h)

Tetrakistriphenylphosphine palladium (0.576 g, 0.5 mmol) was added to a stirred mixture of the 2(tributylstannyl) furan, thiophene, or selenophene 1a-c (10 mmol) and the bromo arylbenzonitrile 2a-e (10 mmol) in de-aireated dioxane (25 mL) under nitrogen. The reaction flask was heated at 100 °C for 12 h. The solvent was concentrated under reduced pressure, the resulting paste was stirred with diethyl ether and filtered. The solid obtained was purified by column chromatography on silica gel, using hexanes/ethyl acetate.

The following compounds was previously reported 4-(furan-2-yl)benzonitrile 3a [27], 6-(furan-2-yl)nicotinonitrile 3b [28], 5-(furan-2-yl)picolinonitrile 3c [28], 4-(thiophen-2-yl)benzonitrile 3d [27], 6-(thiophen-2-yl)nicotinonitrile 3e [28], 4-(selenophen-2-yl)benzonitrile 3f [29].

#### 4.2.2 2-(2-Methyl-4-cyanophenyl) furan (3g)

Orange solid, yield (1.59 g, 87%). mp 42–43 °C; ^1^HNMR (CDCl_3_) δ 7.85 (d, 1H, *J* = 8.0Hz), 7.59 (brs, 1H), 7.56–7.53 (m, 2H), 6.74 (d, 1H, *J* = 3.2Hz), 6.58 (d, 1H, *J* = 3.2Hz), 2.55 (s, 3H); ^13^CNMR (CDCl_3_) δ 149, 134.1, 133.8, 133, 129.5, 126.6, 123.4, 118.9, 113.5, 113.1, 110.4, 21.3; ESI-MS: *m*/*z* calculated for C_12_H_9_NO: 183.21, found: 184.1 (M^+^+1); Anal. Calcd. For C_12_H_9_NO: C, 78.67; H, 4.95; N, 7.65. Found: C, 78.51; H, 5.05; N, 7.43.

#### 4.2.3 2-(2-Methoxy-4-cyanophenyl) furan (3h)

White solid, yield (1.61 g, 81%). mp 54–54.5 °C; ^1^HNMR (CDCl_3_) δ 7.95 (dd, 1H, *J* = 8.0Hz, 2Hz), 7.54 (brs, 1H), 7.46 (d, 1H, *J* = 8Hz), 7.19 (brs, 1H), 7.13 (brs, 1H), 6.56 (dd, 1H, *J* = 3.2Hz, 2.0Hz), 4 (s, 3H); ^13^CNMR (CDCl_3_) δ 149.2, 143.1, 137, 134.6, 130.8, 130.1, 129.6, 127.7, 118.6, 113.6, 111.2, 53.2; ESI-MS: *m*/*z* calculated for C_12_H_9_NO_2_: 199.2, found: 201.1 (M^+^+2); Anal. Calcd. For C_12_H_9_NO_2_: C, 72.35; H, 4.55; N, 7.03. Found: C, 72.41; H, 4.71; N, 6.97.

#### 4.2.4 Synthesis of 2-Bromo-5-(4-cyanoaryl)furan, thiophene or selenophene (4a-h)

*N*-bromosuccinimide (2.13 g, 12 mmol) was added portionwise to a stirred solution of the nitriles 3a-h (10 mmol) in dimethylformamide (20 ml) in an ice bath. The reaction mixture was stirred overnight at room temperature then poured onto ice water, the obtained precipitate was collected and dried. Purification by column chromatography on silica gel, using hexanes/ethyl acetate.

The following compounds was previously reported 4-(5-bromofuran-2-yl)benzonitrile 4a [27], 6-(5-bromofuran-2-yl)nicotinonitrile 4b [28], 5-(5-bromofuran-2-yl)picolinonitrile 4c [28], 4-(5-bromothiophen-2-yl)benzonitrile 4d [27], 6-(5-bromothiophen-2-yl)nicotinonitrile 4e [28], 6-(5-bromoselenophen-2-yl)nicotinonitrile 4f [29].

#### 4.2.5 2-Bromo-5-(2-methyl-4-cyanoaryl) furan (4g)

White solid, yield (2.25 g, 91%). mp 86–88 °C; ^1^HNMR (CDCl_3_) δ 7.82 (d, 1H, *J* = 8.8Hz), 7.56 (d, 2H, *J* = 7.6Hz), 6.69 (d, 1H, *J* = 3.2Hz), 6.49 (d, 1H, *J* = 3.2Hz), 2.53 (s, 3H); ^13^CNMR (CDCl_3_) δ 153.5, 134.9, 134.8, 133, 129.8, 126.8, 123.4, 118.8, 113.7, 113.6, 110.8, 21.9; ESI-MS: *m*/*z* calculated for C_12_H_8_BrNO: 262.1, found: 263.1 (M^+^+1); Anal. Calcd. For C_12_H_8_BrNO: C, 54.99; H, 3.08; N, 5.34. Found: C, 55.07; H, 3.02; N, 5.18.

#### 4.2.6 2-Bromo-5-(2-methoxy-4-cyanoaryl) furan (4h)

Yellow solid, yield (2.41 g, 87%). mp 71–71.5 °C; ^1^HNMR (CDCl_3_) δ 7.89 (brs, 1H), 7.33–7.19 (m, 3H), 6.47 (brs, 1H), 3.99 (s, 3H); ^13^CNMR (CDCl_3_) δ 155.1, 150.9, 126, 125.3, 122.7, 119.1, 116, 115.8, 115.7, 114.9, 111, 56.8; ESI-MS: *m*/*z* calculated for C_12_H_8_BrNO_2_: 278.1, found: 279.2 (M^+^+1); Anal. Calcd. For C_12_H_8_BrNO_2_: C, 51.83; H, 2.90; N, 5.04. Found: C, 51.59; H, 2.76; N, 5.14.

#### 4.2.7 General procedure for the synthesis of the dinitriles 6a-k

Tetrakistriphenylphosphine palladium (0.288 g, 0.25 mmol) was added to a stirred mixture of the indole stannane 5a,b (5 mmol) and the bromo compound 4a-k (5 mmol) in de-aireated dioxane (20 mL) under a nitrogen atmosphere. The reaction mixture was refluxed for 24 h. The solvent was concentrated under reduced pressure, hexanes (20 ml) was added and the mixture was stirred for 2h and filtered. The obtained solid was purified by column chromatography on silica gel, using hexanes/ethyl acetate (75/25, v/v).

1-(*tert*-Butoxycarbonyl)-2-(5-(5-cyanopyridin-2-yl)furan-2-yl)-1*H*-indole-5-carbonitrile (6b). White solid, yield (1.57 g, 77%). mp > 300 °C; ^1^HNMR (DMSO‑*d*_6_) δ 9.03 (s, 1H), 8.37 (d, 1H, *J* = 8.4Hz), 8.25 (brs, 2H), 8.24 (d, 1H, *J* = 3.6Hz), 7.98 (d, 1H, *J* = 8.8Hz), 7.52 (s, 1H), 7.22 (s, 1H), 7.08 (s, 1H), 1.27 (s, 9H);^13^CNMR (DMSO‑*d*_6_) δ 153.4, 152.5, 150.9, 148.9, 148.3, 141.4, 139.1, 131, 128.9, 128.7, 126.9, 119.8, 118.6, 117.6, 116.3, 114.5, 114.1, 112, 107.3, 106.3, 85.7, 27.6; ESI-MS: *m*/*z* calculated for C_24_H_18_N_4_O_3_: 410.42, found: 411.2 (M^+^+1); Anal. Calcd. For C_24_H_18_N_4_O_3_: C, 70.23; H, 4.42; N, 13.65. Found: C, 70.50; H, 4.64; N, 13.44.

1-(*tert*-butoxycarbonyl)-2-(5-(4-cyanophenyl)thiophene-2-yl)-1*H*-indole-5-carbonitrile (6c). White solid, yield (1.65 g, 81%). mp > 300 °C; ^1^HNMR (CDCl_3_) δ 8.56 (s, 1H), 7.74–7.71 (m, 4H), 7.66 (d, 1H, *J* = 8.0Hz), 7.53 (dd, 1H, *J* = 8.0 Hz, 1.2Hz), 7.44 (d, 1H, *J* = 4.0Hz), 7.21 (d, 1H, *J* = 4.0Hz), 6.81 (s, 1H), 1.58 (s, 9H); ^13^CNMR (CDCl_3_) δ 149.9, 149, 141, 140.3, 139.1, 136.5, 132.4, 131.6, 128.6, 127.1, 126.7, 126.6, 125.3, 119.5, 118.3, 116.5, 111.1, 111, 106.1, 86.1, 27.9; ESI-MS: *m*/*z* calculated for C_25_H_19_N_3_O_3_: 409.44, found: 410.2 (M^+^+1); Anal. Calcd. For C_25_H_19_N_3_O_3_: C, 73.43; H, 4.68; N, 10.26. Found: C, 73.02; H, 4.72; N, 10.15.

1-(*tert*-butoxycarbonyl)-2-(5-(4-cyanophenyl)selenophen-2-yl)-1*H*-indole-5-carbonitrile (6d). White solid, yield (1.65 g, 70%). mp > 300 °C; ^1^HNMR (CDCl_3_) δ 8.28 (d, 1H, *J* = 8.8Hz), 7.9 (brs, 2H), 7.70–7.67 (m, 3H), 7.61 (s, 1H), 7.59 (d, 1H, *J* = 4.0Hz), 7.35 (d, 1H, *J* = 4.0Hz), 6.79 (s, 1H), 1.52 (s, 9H); ^13^CNMR (CDCl_3_) δ 150, 149.2, 141, 140.2, 139.2, 136.5, 132.9, 131.7, 128.9, 127.9, 126.9, 126.5, 125.5, 119.6, 118.7, 116.3, 111.2, 111.1, 106.7, 85.5, 27.8; ESI-MS: *m*/*z* calculated for C_25_H_19_N_3_O_2_Se: 472.4, found: 473.3 (M^+^+1); Anal. Calcd. For C_25_H_19_N_3_O_2_Se: C, 63.56; H, 4.05; N, 8.90. Found: C, 63.33; H, 3.96; N, 8.84.

1-(*tert*-butoxycarbonyl)-2-(5-(5-cyanopyridin-2-yl)furan-2-yl)-1*H*-indole-6-carbonitrile (6f). White solid, yield (1.25 g, 61%). mp > 300 °C; ^1^HNMR (CDCl_3_) δ 8.87 (brs, 1H), 8.59 (brs, 1H), 8.01 (dd, 1H, *J* = 8.4Hz, 1.6Hz), 7.82 (d, 1H, *J* = 8.0Hz), 7.69 (d, 1H, *J* = 8Hz), 7.55 (dd, 1H, *J* = 8.4Hz, 1.6Hz), 7.38 (d, 1H, *J* = 3.6Hz), 6.96 (brs, 1H), 6.88 (d, 1H, *J* = 3.6Hz), 1.5 (s, 9H); ^13^CNMR (CDCl_3_) δ 152.1, 152, 150.8, 148.3, 148, 141.4, 139, 131.2, 129, 128.7, 126.9, 119.7, 118.3, 117.2, 116.2, 114.4, 114.1, 112, 107.1, 106.4, 85.4, 27.6; ESI-MS: *m*/*z* calculated for C_24_H_18_N_4_O_3_: 410.42, found: 411.1 (M^+^+1); Anal. Calcd. For C_24_H_18_N_4_O_3_: C, 70.23; H, 4.42; N, 13.65. Found: C, 69.91; H, 4.58; N, 13.33.

1-(*tert*-butoxycarbonyl)-2-(5-(6-cyanopyridin-3-yl)furan-2-yl)-1*H*-indole-6-carbonitrile (6g). White solid, yield (1.41 g, 69%). mp > 300 °C; ^1^HNMR (DMSO‑*d*_6_) δ 9.21 (d, 1H, *J* = 2.0 Hz), 8.45 (brs, 1H), 8.93 (dd, 1H, *J* = 8.0 Hz, 2Hz), 8.13 (d, 1H, *J* = 8.0 Hz), 7.9 (d, 1H, *J* = 8.0 Hz), 7.72 (d, 1H, *J* = 8.0 Hz), 7.6 (d, 1H, *J* = 3.6 Hz), 7.28 (s, 1H), 7.12 (d, 1H, *J* = 3.6 Hz), 1.42 (s, 9H); ^13^CNMR (DMSO‑*d*_6_) δ 149.7, 148.1, 146.3, 136.4, 132.1, 131.7, 130.6, 128.9, 128.6, 126.4, 121.8, 120.2, 119.8, 117.3, 113.5, 111.7, 111, 108.2, 85.5, 27.5; ESI-MS: *m*/*z* calculated for C_24_H_18_N_4_O_3_: 410.42, found: 411.1 (M^+^+1); Anal. Calcd. For C_24_H_18_N_4_O_3_: C, 70.23; H, 4.42; N, 13.65. Found: C, 69.90; H, 4.38; N, 13.40.

1-(*tert*-butoxycarbonyl)-2-(5-(4-cyano-2-methylphenyl)furan-2-yl)-1*H*-indole-6-carbonitrile (6h). Yellow solid, yield (1.37 g, 65%). mp > 300 °C; ^1^HNMR (CDCl_3_) δ 8.57 (s, 1H), 7.66 (d, 1H, *J* = 8.4Hz), 7.61 (brs, 1H), 7.55 (brs, 2H), 7.54 (dd, 1H, *J* = 8.4 Hz, 1.6Hz), 7.21 (d, 1H, *J* = 4.0Hz), 7.17 (d, 1H, *J* = 4.0Hz), 6.81 (s, 1H), 2.56 (s, 3H), 1.52 (s, 9H); ^13^CNMR (CDCl_3_) δ 149.2, 142.3, 138.2, 137, 136.5, 135.3, 134.6, 131.9, 130.7, 129.8, 128.7, 127.4, 126.3, 123.6, 121.4, 120.1, 120, 118.7, 111.9, 111.5, 107.6, 85.3, 27.75, 21.3; Anal. Calcd. For C_26_H_21_N_3_O_3_: C, 73.74; H, 5.00; N, 9.92. Found: C, 73.74.; H, 5.09.; N, 9.78.

1-(*tert*-butoxycarbonyl)-2-(5-(4-cyano-2-methoxyphenyl)furan-2-yl)-1*H*-indole-6-carbonitrile (6i). Yellow solid, yield (1.33 g, 61%). mp > 300 °C; ^1^HNMR (DMSO‑*d*_6_) δ 8.44 (s, 1H), 7.97 (d, 1H, *J* = 8.0Hz), 7.89 (d, 1H, *J* = 8.0Hz), 7.71 (d, 1H, *J* = 8.0Hz), 7.65 (brs, 1H), 7.53 (d, 1H, *J* = 8.0Hz), 7.31–7.30 (m, 1H), 7.24 (s, 1H), 7.06–7.05 (m, 1H), 4.04 (s, 3H), 1.36 (s, 9H); ESI-MS: *m*/*z* calculated for C_26_H_21_N_3_O_4_: 439.46, found: 440.4 (M^+^+1); Anal. Calcd. For C_26_H_21_N_3_O_4_: C, 71.06; H, 4.82; N, 9.56. Found: C, 71.22; H, 4.83; N, 9.49.

1-(*tert*-butoxycarbonyl)-2-(5-(4-cyanophenyl)thiophen-2-yl)-1*H*-indole-6-carbonitrile (6j). Yellow solid, yield (1.44 g, 68%). mp > 300 °C; ^1^HNMR (CDCl_3_) δ 8.57 (brs, 1H), 7.74–7.69 (m, 4H), 7.64 (d, 1H, *J* = 8.0Hz), 7.54 (dd, 1H, *J* = 8.4 Hz, 1.6Hz), 7.43 (d, 1H, *J* = 4Hz), 7.2 (d, 1H, *J* = 4Hz), 6.82 (s, 1H), 1.51 (s, 9H); ^13^CNMR (CDCl_3_, 400 MHz) δ 149.1, 143.2, 138, 136.5, 135.7, 135.5, 132.9, 131.9, 129.7, 126.3, 126, 124.8, 121.5, 120.1, 120, 118.7, 111.9, 111, 107.7, 85.5, 27.7; ESI-MS: *m*/*z* calculated for C_25_H_19_N_3_O_2_S: 425.5, found: 426.2 (M^+^+1); Anal. Calcd. For C_25_H_19_N_3_O_2_S: C, 70.57; H, 4.50; N, 9.88. Found: C, 70.91; H, 4.57; N, 9.62.

1-(*tert*-butoxycarbonyl)-2-(5-(5-cyanopyridin-2-yl)thiophen-2-yl)-1*H*-indole-6-carbonitrile (6k). brown solid, yield (1.32 g, 62%). mp > 300 °C; ^1^HNMR (CDCl_3_) δ 8.84 (d, 1H, *J* = 1.6Hz), 8.55 (brs, 1H), 7.99 (dd, 1H, *J* = 8.4 Hz, 2Hz), 7.78 (d, 1H, *J* = 8.0Hz), 7.7 (d, 1H, *J* = 3.6Hz), 7.67 (d, 1H, *J* = 8.0Hz), 7.55 (dd, 1H, *J* = 8.0Hz, 1.6Hz), 7.24 (d, 1H, *J* = 3.6Hz), 6.83 (s, 1H), 1.49 (s, 9H); ^13^CNMR (CDCl_3_) δ 149.6, 148.1, 148, 146.3, 136.4, 132, 131.7, 130.7, 128.8, 128.6, 126.3, 121.4, 120.2, 119.8, 117.2, 113.4, 111.7, 111, 108.2, 85.4, 27.9; ESI-MS: *m*/*z* calculated for C_24_H_18_N_4_O_2_S: 426.49, found: 427.5 (M^+^+1); Anal. Calcd. For C_24_H_18_N_4_O_2_S: C, 67.59; H, 4.25; N, 13.14. Found: C, 67.84; H, 4.33; N, 12.99.

#### 4.2.8 General procedure for the synthesis of the diamidines 7a-k

The dinitriles 6a-k (0.66 mmol) was suspended in freshly distilled THF (5 ml), and treated with lithium trimethylsilylamide 1 M solution in tetrahydrofuran (4 ml, 3.98 mmol), the mixture was stirred for 2 days at room temperature. The reaction mixture was then cooled to zero ^o^C and HCl saturated ethanol (2 ml) was added. The mixture was stirred for 24 h, diluted with dry diethyl ether and the produced solid was collected by filtration. The diamidine was purified by neutralization with 1 *N* sodium hydroxide solution followed by filtration of the formed solid and washing with water and dried. Finally, the free base was stirred with ethanolic HCl for one week to make sure that the (Boc)_2_O group was completely removed, diluted with dry diethyl ether, and the solid formed was filtered and dried to give the diamidines hydrochloride salt.

#### 4.2.9 2-(5-(5-Amidinopyridine-2-yl)) furan-2-yl)-1*H*-indole-5-amidine (7b)

Yellow solid, yield (0.188 g, 62%), mp > 300 °C; ^1^HNMR (DMSO‑*d*_6_) δ 12.65 (s, 1H), 9.68 (s, 2H), 9.37 (s, 2H), 9.29 (s, 2H), 9.05 (s, 2H), 8.42 (d, 1H, *J* = 8.4Hz), 8.23–8.20 (m, 2H), 7.64 (brs, 2H), 7.54–7.52 (m, 2H), 7.33–7.31 (m, 1H), 7.18 (s, 1H); ^13^CNMR (DMSO‑*d*_6_) δ 166.9, 164, 152, 149.7, 149.5, 140.2, 137.8, 131.2, 128.2, 122.4, 122.1, 119.6, 118.4, 114.8, 112.3, 110.8, 100.8; ESI-MS: *m*/*z* calculated for C_19_H_16_N_6_O: 344.37, found: 345.5 (amidine base M^+^+1); Anal. Calcd. For C_19_H_16_N_6_O . 3HCl. 0.4H_2_O: C, 49.5; H, 4.32; N, 18.23. Found: C, 49.8; H, 4.44; N, 18.04.

#### 4.2.10 2-(5-(4-Amidinophenyl) thiophene-2-yl)-1*H*-indole-5-amidine (7c)

Yellow solid, yield (0.151 g, 52%), mp > 300 °C; ^1^HNMR (DMSO‑*d*_6_) δ 12.51 (s, 1H), 9.44 (s, 2H), 9.24 (s, 2H), 9.18 (s, 2H), 8.95 (s, 2H), 8.14 (br s, 1H), 7.97–7.94 (m, 4H), 7.85 (d, 1H, *J* = 3.6Hz), 7.77 (d, 1H, *J* = 3.6Hz), 6.98 (brs, 1H); ESI-MS: *m*/*z* calculated for C_20_H_17_N_5_S: 359.45, found: 360.4 (amidine base M^+^+1); Anal. Calcd. For C_20_H_17_N_5_S . 2HCl. 1H_2_O: C, 53.44; H, 4.71; N, 15.59. Found: C, 53.37; H, 4.89; N, 15.22.

#### 4.2.11 2-(5-(4-Amidinophenyl) selenophen-2-yl)-1*H*-indole-5-amidine (7d)

Yellow solid, yield (0.149 g, 45%), mp > 300 °C; ^1^HNMR (DMSO‑*d*_6_) δ12.55 (s, 1H), 9.49 (s, 2H), 9.27 (brs, 4H), 9.02 (s, 2H), 8.14 (s, 1H), 7.99 (d, 1H, *J* = 3.6Hz), 7.95–7.92 (m, 4H), 7.9 (d, 1H, *J* = 8.4Hz), 7.60–7.57 (m, 2H), 6.98 (d, 1H, *J* = 1.6Hz); ^13^CNMR (DMSO‑*d*_6_) δ 166.9, 165.3, 147.3, 141.1, 140.7, 140.4, 136.7, 129.8, 129.6, 128.9, 128.5, 126.9, 126.1, 121.7, 119.5, 112.1, 101.8; ESI-MS: *m*/*z* calculated for C_20_H_17_N_5_Se: 406.34, found: 407.5 (amidine base M^+^+1); Anal. Calcd. For C_20_H_17_N_5_Se. 3HCl. H_2_O. 0.25Et_2_O: C, 48.99; H, 4.83; N, 17.14. Found: C, 49.04; H, 4.51; N, 16.89.

#### 4.2.12 2-(5-(5-Amidinopyridine-2-yl)) furan-2-yl)-1*H*-indole-6-amidine (7f)

Yellow solid, yield (0.19 g, 59%), mp > 300 °C; ^1^HNMR (DMSO‑*d*_6_) δ12.68 (s, 1H), 9.69 (s, 2H), 9.47 (brs, 4H), 9.1 (s, 2H), 8.45–8.43 (m, 2H), 8.26 (d, 1H, *J* = 8.0Hz), 7.91 (s, 1H), 7.72 (d, 1H, *J* = 8.0Hz), 7.58 (d, 1H, *J* = 3.6Hz), 7.51 (d, 1H, *J* = 8.0Hz), 7.47 (d, 1H, *J* = 3.6Hz), 7.2 (s, 1H),; ^13^CNMR (DMSO‑*d*_6_) δ 166.9, 164, 152, 149.8, 149.5, 140.3, 137.9, 131.2, 128.2, 122.3, 122.1, 119.5, 118.4, 114.8, 112.3, 110.8, 100.8; ESI-MS: *m*/*z* calculated for C_19_H_16_N_6_O: 344.37, found: 345.3 (amidine base M^+^+1); Anal. Calcd. For C_19_H_16_N_6_O +3HCl+1H_2_O+0.25Et_2_O: C, 48.99; H, 4.83; N, 17.14. Found: C, 49.04; H, 4.51; N, 16.89.

#### 4.2.13 2-(5-(6-Amidinopyridine-3-yl)) furan-2-yl)-1*H*-indole-6-amidine (7g)

Yellow solid, yield (0.163 g, 51%), mp > 300 °C; ^1^HNMR (DMSO‑*d*_6_) δ12.6 (s, 1H), 9.62 (s, 2H), 9.41 (s, 2H), 9.34 (brs, 2H), 9 (s, 2H), 8.63 (s, 1H), 8.49 (brs, 1H), 7.97 (s, 1H), 7.77 (brs, 1H), 7.64 (d, 1H, *J* = 3.2Hz), 7.48 (brs, 1H), 7.36 (d, 1H, *J* = 3.2Hz), 7.14 (brs, 2H); ^13^CNMR (DMSO‑*d*_6_) δ 166.8, 161.8, 149.7, 148.9, 145.3, 142.2, 136.7, 132.7, 132.6, 130, 124.2, 121.4, 121, 119.6, 113.7, 112.6, 111.2, 100.2; ESI-MS: *m*/*z* calculated for C_19_H_16_N_6_O: 344.37, found: 345.3 (amidine base M^+^+1); Anal. Calcd. For C_19_H_16_N_6_O .2HCl.2.5H_2_O.0.5EtOH: C, 49.49; H, 5.39; N, 17.31. Found: C, 49.17; H, 5.12; N, 17.06.

##### 4.2.13.1 2-(5-(4-Amidino-2-methylphenyl) furan-2-yl)-1*H*-indole-6-amidine (7h)

Yellow solid, yield (0.158 g, 49%), mp > 300 °C; ^1^HNMR (DMSO‑*d*_6_) δ 12.5 (s, 1H), 9.44 (s, 2H), 9.3 (s, 2H), 9.19 (s, 2H), 8.97 (s, 2H), 7.92 (brs, 1H), 7.88 (brs, 1H), 7.83 (brs, 1H), 7.76–7.72 (m, 3H), 7.51 (brs, 1H), 7.47–7.45 (m, 1H), 6.93 (s, 1H), 2.51 (s, 3H); ^13^CNMR (DMSO‑*d*_6_) δ 167.4, 166, 141, 138.7, 136.7, 136.6, 136.1, 133.2, 131.2, 130.4, 129.6, 127.6, 126.6, 126.4, 123.5, 121, 120.6, 119.4, 112.5, 100.3, 22; ESI-MS: *m*/*z* calculated for C_21_H_19_N_5_O: 357.41, found: 358.3 (amidine base M^+^+1); Anal. Calcd. For C_21_H_19_N_5_O. 2HCl. 2.1H_2_O. 0.5EtOH: C, 53.79; H, 5.78; N, 14.25. Found: C, 53.58; H, 5.70; N, 14.49.

#### 4.2.14 2-(5-(4-Amidino-2-methoxyphenyl)furan-2-yl)-1*H*-indole-6-amidine (7i)

Yellow solid, yield (0.184 g, 33%), mp > 300 °C; ^1^HNMR (DMSO‑*d*_6_) δ 12.7 (s, 1H), 9.61 (s, 2H), 9.51 (s, 2H), 9.47 (s, 2H), 9.36 (s, 2H), 8.42 (d, 1H, *J* = 8Hz), 8.02 (s, 1H), 7.76 (d, 1H, *J* = 8Hz), 7.69–7.65 (m, 2H), 7.49 (d, 1H, *J* = 8Hz), 7.34–7.32 (m, 2H), 7.1 (s, 1H), 4.09 (s, 3H); ^13^CNMR (DMSO‑*d*_6_) δ 166.9, 165.1, 155.2, 148.8, 147.1, 136.3, 133.1, 132.8, 127.4, 126.3, 125.9, 123.3, 121, 120.9, 119.5, 115.8, 112.5, 112, 111.2, 99.7, 56.7; ESI-MS: *m*/*z* calculated for C_21_H_19_N_5_O_2_: 373.41, found: 374.4 (amidine base M^+^+1); Anal. Calcd. For C_21_H_19_N_5_O_2_. 2HCl. 1.75H_2_O. 0.15EtOH: C, 52.77; H, 5.28; N, 14.44. Found: C, 52.42; H, 5.5.34; N, 14.39.

#### 4.2.15 2-(5-(4-Amidinophenyl) thiophen-2-yl)-1*H*-indole-6-amidine (7j)

Yellow solid, yield (0.122 g, 41%), mp > 300 °C; ^1^HNMR (DMSO‑*d*_6_) δ12.55 (s, 1H), 9.63 (s, 2H), 9.48 (brs, 4H), 9.04 (brs, 2H), 8.33 (brs, 2H), 8.25 (d, 1H, *J* = 8.8Hz), 8.21 (d, 1H, *J* = 8.0Hz), 7.94 (s, 1H), 7.87 (d, 1H, *J* = 3.6Hz), 7.76 (d, 1H, *J* = 8.4Hz), 7.48 (d, 1H, *J* = 8.4Hz), 7.02 (s, 1H); ^13^CNMR (DMSO‑*d*_6_) δ 167, 165, 152, 148, 136.1, 134.9, 133, 132.4, 129.5, 126.6, 123.9, 121, 120.7, 119.6, 112.3, 111.8, 110.7, 99.7; ESI-MS: *m*/*z* calculated for C_20_H_17_N_5_S: 359.45, found: 360.2 (amidine base M^+^+1); Anal. Calcd. For C_20_H_17_N_5_S .2HCl.1.25H_2_O: C, 52.80; H, 4.76; N, 15.39. Found: C, 52.44; H, 4.82; N, 15.11.

#### 4.2.16 2-(5-(5-Amidinopyridine-2-yl))thiophen-2-yl)-1*H*-indole-6-amidine (7k)

Yellow solid, yield (0.208 g, 61%), mp > 300 °C; ^1^HNMR (DMSO‑*d*_6_) δ12.65 (s, 1H), 9.54 (s, 2H), 9.47 (s, 2H), 9.43 (s, 2H), 9.37 (s, 2H), 7.98–7.88 (m, 5H), 7.78 (brs, 2H), 7.38 (d, 1H, *J* = 8.4Hz), 7.05 (d, 1H, *J* = 8.4Hz), 6.92 (s, 1H); ^13^CNMR (DMSO‑*d*_6_) δ 166.8, 161.8, 149.5, 148.9, 145.2, 142.1, 136.3, 132.7, 132, 129.2, 124, 122, 121.3, 119.6, 113.7, 112.6, 111.3, 100.2; ESI-MS: *m*/*z* calculated for C_19_H_16_N_6_S: 360.44, found: 360.3 (amidine base M^+^+1); Anal. Calcd. For C_19_H_16_N_6_S .3HCl.2H_2_O.0.25EtOH: C, 45.26; H, 4.77; N, 16.24. Found: C, 45.42; H, 4.89; N, 16.20.

## Declaration of competing interest

The authors declare the following financial interests/personal relationships which may be considered as potential competing interests:

## Data Availability

No data was used for the research described in the article.
